# Transport von schwer verletzten Traumapatienten im Rettungswagen mit und ohne starre Halsorthese: vergleichende biomechanische Messungen

**DOI:** 10.1007/s00101-024-01462-w

**Published:** 2024-09-24

**Authors:** Martin Kieninger, Corinna Schneider, Simon Auer, Lukas Reinker, Ina Adler, Sebastian Dendorfer, Johanna Rosenberger, Daniel Popp, Christoph Eissnert, Dominik Ludsteck, Christopher Cyrus, Johannes Hoffmann, Sarah Morag, Bernhard Graf, Bärbel Kieninger

**Affiliations:** 1 Klinik für Anästhesiologie, Universitätsklinikum Regensburg, Regensburg, Deutschland; 2 Labor für Biomechanik, Ostbayerische Technische Hochschule (OTH) Regensburg, Regensburg, Deutschland; 3 Klinik und Poliklinik für Unfallchirurgie, Universitätsklinikum Regensburg, Regensburg, Deutschland; 4 Malteser Hilfsdienst e. V., Rettungswache Regensburg, Regensburg, Deutschland; 5 Abteilung für Krankenhaushygiene und Infektiologie, Universitätsklinikum Regensburg, Regensburg, Deutschland; 6 Klinik und Poliklinik für Herz‑, Thorax- und herznahe Gefäßchirurgie, Universitätsklinikum Regensburg, Regensburg, Deutschland

**Keywords:** Halswirbelsäule, Zervikalstütze, Vakuummatratze, Immobilisation, Notfallmedizin, Cervical spine, Cervical collar, Vacuum mattress, Immobilization, Emergency medical care

## Abstract

**Hintergrund:**

Der tatsächliche Stellenwert der prähospitalen Immobilisation der Halswirbelsäule (HWS) bei schwer verletzten Traumapatienten ist weiterhin unklar. In Anbetracht möglicher negativer Implikationen durch das Anbringen einer starren HWS-Orthese muss deren Anwendung während der gesamten prähospitalen Phase kritisch hinterfragt werden.

**Ziel der Arbeit:**

Es sollten vergleichende biomechanische Messungen zur Beweglichkeit der HWS bei Immobilisation mittels Vakuummatratze mit und ohne zusätzliches Anbringen einer starren HWS-Orthese nach Lagerung auf der Trage durchgeführt werden.

**Material und Methoden:**

Die Bewegungen der HWS beim Ein- und Ausladeprozess in einen modernen RTW sowie während der Fahrt entlang eines vordefinierten Parkours wurden mit einem Motion-Capture-System aufgezeichnet. Die Probandin, an der die Messungen durchgeführt wurden, wurde auf einer Vakuummatratze mit der Möglichkeit zur seitlichen Fixierung des Kopfes sowie Kinn- und Stirngurt auf einer elektrohydraulischen Fahrtrage immobilisiert. Bei der einen Hälfte der Versuche erfolgte die zusätzliche Immobilisation der HWS mittels starrer Orthese, bei der anderen wurde auf die Anwendung einer HWS-Orthese verzichtet.

**Ergebnisse:**

Statistisch signifikante Unterschiede ergaben sich nur bei einigen biomechanischen Parametern in der sagittalen Ebene (Flexion und Extension). Für die anderen Bewegungsrichtungen (axiale Rotation, laterale Beugung) wurden keine signifikanten Unterschiede für die gemessenen Parameter ermittelt. Generell wurden sowohl bei den Versuchen mit HWS-Orthese als auch ohne HWS-Orthese nur sehr geringe Winkelauslenkungen (im Mittel bei axialer Rotation und Flexion/Extension im Bereich von einem bis 2 Grad, bei der lateralen Beugung höchstens 3 Grad) gemessen.

**Schlussfolgerung:**

Bei einer korrekt durchgeführten Immobilisation mittels einer Vakuummatratze mit der Möglichkeit zur seitlichen Stabilisierung des Kopfes sowie Kinn- und Stirngurt auf einer elektrohydraulischen Fahrtrage mit Beladesystem ergeben sich für den Ein- und Ausladeprozess sowie während der Fahrt in einem modernen RTW mit luftgefederter Tragenlagerung und Luftfederung der Hinterachse keine relevanten Vorteile bezüglich der Einschränkung der Bewegung der HWS durch die zusätzliche Verwendung einer starren HWS-Orthese.

## Abstract (en)

**Background:**

The actual significance of prehospital immobilization of the cervical spine in severely injured trauma patients remains unclear. In view of possible negative implications, such as an increase in intracranial pressure due to the application of a rigid cervical spine orthosis, the long-term use must be critically questioned. Further studies are required to justify the long-term use of a rigid cervical spine orthosis in the prehospital setting.

**Objective:**

Comparative measurements of the mobility of the cervical spine during immobilization using a vacuum mattress with or without the additional application of a rigid cervical spine orthosis after positioning on the stretcher were carried out.

**Material and methods:**

Biomechanical measurements of the movement of the cervical spine were carried out by attaching inertial measurement units to a test person during the loading and unloading process in a modern ambulance and during the journey along a predefined parkour. The test person on whom the measurements were carried out was immobilized on a vacuum mattress with the option of lateral fixation of the head and chin and forehead strap on an electrohydraulic stretcher. The complete standard monitoring was set up to simulate as realistic a transport of a severely injured patient as possible. A total of 30 test runs were realized. In one half of the tests, the cervical spine was additionally immobilized using a rigid orthosis and in the other half a cervical spine orthosis was not used. For each of the 30 tests, the angles, axial rotation, lateral bending and flexion/extension as well as the first and second derivatives were considered for loading, transport and unloading and the parameters mean deviation from the zero position, size of the swept angle range and maximum were calculated for each test run.

**Results:**

Statistically significant differences were only found for some biomechanical parameters in the sagittal plane (flexion and extension). No significant differences were found for the measured parameters in the other directions of movement (axial rotation, lateral flexion). In general, only very small angular deflections were measured both in the tests with the cervical spine orthosis and without the cervical spine orthosis (on average in the range of 1–2° for axial rotation and flexion/extension and up to 3° for lateral flexion).

**Conclusion:**

If immobilization is carried out correctly using a vacuum mattress with the option of lateral stabilization of the head and chin and a forehead strap on an electrohydraulic stretcher with a loading system, there are no relevant advantages with respect to the restriction of movement of the cervical spine by the additional use of a rigid cervical spine orthosis for the loading and unloading process or during the transport in a modern ambulance. It could therefore be advantageous to remove the rigid cervical spine orthosis initially applied for the rescue of the patient at the scene after the patient has been positioned on the vacuum mattress and stretcher to avoid potential negative effects of the rigid cervical spine orthosis for the period of transportation to the hospital.

## Hintergrund

Bei schwer verletzten Patienten finden sich häufig auch Verletzungen der Wirbelsäule. In einer europaweiten Studie wurde ermittelt, dass 13 % der Patienten, die ein schweres Trauma erleiden, auch von einem Wirbelsäulentrauma betroffen sind, in 45 % der Fälle an der Halswirbelsäule (HWS) [4]. In Arbeiten basierend auf Daten aus Deutschland wurde eine Beteiligung der Wirbelsäule bei 17 % bzw. 34 % der Betroffenen ermittelt [1, 12].

Die hohe Inzidenz von Verletzungen der HWS bei schwer verletzten Patienten hat zu einer sehr großzügigen Indikationsstellung für die Immobilisation der HWS mittels starrer Orthesen in der prähospitalen Notfallmedizin geführt. Die Immobilisation der HWS mittels starrer Orthese sollte jedoch aufgrund potenzieller, durch diese Maßnahme bedingter Probleme wie Ansteigen des intrakraniellen Drucks oder Beeinträchtigung der oberen Atemwege und Erschweren der Atemwegssicherung [3, 5–8, 11] sowie häufig gegebener praktischer Schwierigkeiten bei der Anlage infolge langer Haare, störender Kleidungsstücke oder der ungünstigen Positionierung des Patienten nicht unkritisch erfolgen.

Grundsätzlich erscheint es sinnvoll, die Zeitdauer der Immobilisation der HWS durch eine starre Orthese in der prähospitalen Phase so kurz wie möglich zu halten.

In der vorliegenden Arbeit wurde untersucht, ob durch die alleinige Verwendung einer Vakuummatratze beim Ein- und beim Ausladeprozess sowie beim Transport in einem modernen Rettungswagen (RTW) der Bewegungsumfang der HWS ähnlich eingeschränkt werden kann wie bei der zusätzlichen Anlage einer starren HWS-Orthese.

## Methode

Die Daten wurden im Rahmen einer explorativen biomechanischen Studie erhoben. Die Studie wurde nach positivem Votum der Ethikkommission des Universitätsklinikums Regensburg (20-1661-101) durchgeführt. Die Probandin nahm freiwillig an der Studie teil und hatte im Vorfeld schriftlich für die Teilnahme eingewilligt.

### Aufbau der Versuchsreihe und Material

**Abb. 1:**
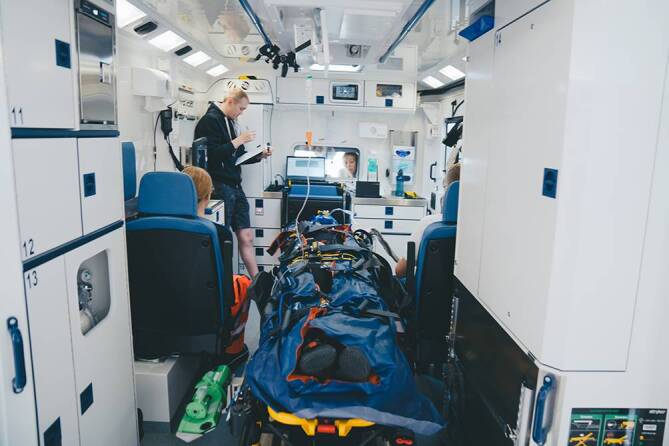
Blick in den Rettungswagen (RTW BY 2022) während der Versuchsdurchführung

Ein kompletter Versuchsablauf bestand aus dem Einladeprozess in den RTW, der Fahrt entlang einer festgelegten Strecke und dem Ausladeprozess. Alle Versuche wurden mit derselben Probandin (weiblich, 22 Jahre, Größe 1,78 m, Gewicht 71 kg, gesund) und von demselben Team durchgeführt, welches aus einer Notärztin, einem Notfallsanitäter und einer Rettungssanitäterin bestand. Die zu fahrende Strecke am Gelände des Universitätsklinikums Regensburg (ca. 800 m) war bei jedem Versuch identisch und beinhaltete mehrere Kurven, Schwellen und Bordsteinkanten. Für die Versuche stand ein moderner RTW des Rettungsdienstes Bayern (RTW BY 2022, Mercedes-Benz Sprinter 519 CDI, Stuttgart, Deutschland; Fa. Wietmarscher Ambulanz- und Sonderfahrzeugbau, Emsbüren, Deutschland; Stryker Power-LOAD elektrohydraulische Fahrtrage mit Beladesystem, Fa. Stryker Kalamazoo, MI, USA; luftgefederte Tragenlagerung, Luftfederung Hinterachse, Abb. 1) zur Verfügung. Bei der verwendeten Vakuummatratze handelte es sich um das Modell Schnitzler Rettungsprodukte 826 K-H+ mit integrierter Kopffixierung sowie Kinn- und Stirngurt (Fa. Schnitzler Rettungsprodukte, Niederkassel-Mondorf, Deutschland). Als starre HWS-Orthese wurde das Modell Ambu perfit ACE (Fa. Ambu, Bad Nauheim, Deutschland) verwendet.

**Abb. 2:**
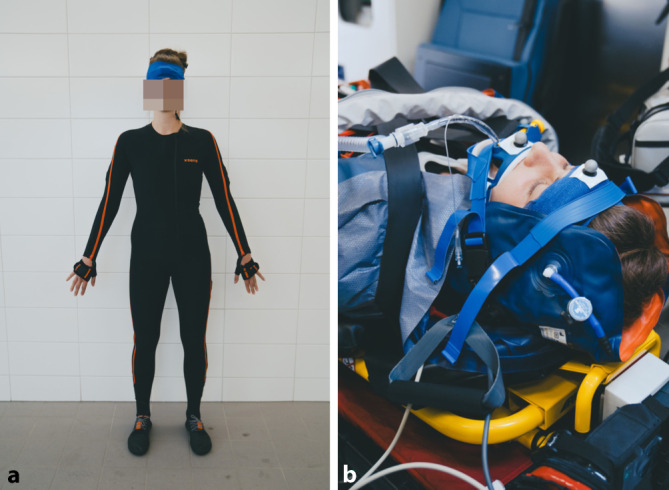
**a** Für die Versuchsdurchführung trägt die Probandin den Anzug Xsens MVN Link (Fa. Xsens Technologies, Enschede, Niederlande), in den 17 Sensoren integriert sind. **b** Immobilisierte Probandin während der Versuchsdurchführung mit angebrachtem Endotrachealtubus

Die Messung der Bewegung in der Halswirbelsäule erfolgte mit dem System Xsens MVN (Fa. Xsens Technologies, Enschede, Niederlande). Die Probandin trug während des Versuchs einen Anzug aus Lycra (Xsens MVN Link, Fa. Xsens Technologies, Enschede, Niederlande), in dem an festen Stellen die Sensoren des Systems befestigt sind; ebenso wurde ein weiterer Sensor an der Stirn der Probandin befestigt (Abb. 2a). Mit diesem System können über Gyroskope, Beschleunigungssensoren und Magnetometer die Bewegungen zueinander und bezüglich der Richtung zum Erdmittelpunkt bestimmt werden. Vor den Messungen wurde eine Kalibrierung des Systems vorgenommen, die auch nach jeweils 3 Versuchen wiederholt wurde. Die Messungen wurden mit einer Frequenz von 240 Hz aufgenommen. Um einen möglichst realistischen Transport eines schwer verletzen Patienten nachzustellen, wurde das komplette Standardmonitoring (Kabel für Elektrokardiogramm, Sensor für Pulsoxymetrie, Blutdruckmanschette) etabliert. Zudem wurde eine Venenverweilkanüle mit angeschlossener Infusion an einer Hand angeklebt und ein gekürzter Endotrachealtubus mit konnektiertem Transportbeatmungsgerät angebracht (Abb. 2b). Es wurden jeweils 15 Versuche mit und 15 Versuche ohne HWS-Orthese durchgeführt.

Der Ablauf jedes Versuchsdurchgangs war bei den Versuchen mit und ohne HWS-Orthese identisch (Tab. 1). Das Antippen des Stirnsensors diente zur Überprüfung des Messsystems. Vor der Berechnung der relevanten Größen wurden deswegen die Daten aus dem Zeitabschnitt M2–M3 entfernt.

**Tab. 1:** Chronologischer Ablauf der Versuche

Zeitabschnitt	Zeitmarker	Handlung
–	M0	–
Kalibrierung	–	Etwa 30 s Bewegungslosigkeit zur Nullwinkelbestimmung
–	M1	–
Einladen	–	F: Lösen der Bremse an der Trage
		F: Einladen der Trage
		B: Einstieg in den RTW über die Hecktür mit Notfallbeatmungsgerät
	M2	–
	–	B: Antippen des Stirnsensors mit freier Hand
	M3	–
	–	NÄ: Einstieg in den RTW über die Hecktür
		B: Verladen des Notfallbeatmungsgeräts
		B: Verladen des Monitors
		B: Verladen der Absaugpumpe
		NÄ: Aufhängen der Infusion
		B: Aktivierung des Fahrmodus an der Trage
		B und NÄ: Ausklappen der Sitze
		F: Schließen der Hecktüre von außen
		F: Einstieg und Schließen der Fahrertür
–	M4	–
Fahrt	–	Fahren der Strecke
		F: Aussteigen
–	M5	–
Ausladen	–	B und NÄ: Einklappen der Sitze
		B: Deaktivierung des Fahrmodus an der Trage
		B: Aufnahme des Monitors, Übergabe an NÄ
		B: Aufnahme des Notfallbeatmungsgeräts
		NÄ: Abhängen der Infusion
		F: Öffnen der Hecktür
		NÄ: Ausstieg über die Hecktür
		F: Ausladen der Trage
		B: Ausstieg über die Hecktür mit Notfallbeatmungsgerät
–	M6	–

*F* Fahrerin, *B* Beifahrer/Notfallsanitäter, *NÄ* Notärztin, *RTW* Rettungswagen

### Mathematische Analyse und Statistik

Die Rohdaten wurden in die Software Xsens MVN (Version 2023.02, Scenario No Level, Fa. Xsens Technologies, Enschede, Niederlande) importiert und auf das durch die Maße der Probandin optimierte Modell übertragen. Die Koordinaten und Winkel dieses Modells wurden in einen Excel-File exportiert und mit einem selbstgeschriebenen Pythonskript (numpy, panda, scipy) weiterverarbeitet. Die Bewegung in der HWS wird durch den Winkel „ergonomic joint angle Head_T8“ repräsentiert, der die Bewegung vom Kopf zum Sternum als Euler-Winkel misst (Verwendung der Repräsentation ZXY mit Z Kraniokaudalachse, X Sagitallachse und Y Horizontalachse). Der Mittelwert des während der Kalibrierung gemessenen Winkels wurde als Nullwinkel definiert. Es wurde eine Transformation der Winkelanteile durchgeführt, sodass diese die Auslenkung bezüglich dieses Nullwinkels über die Zeit repräsentieren. Die Daten wurden im Anschluss mit einem Gauß-Glättungsfilter bearbeitet, um das Signal-Rausch-Verhältnis zu verbessern. Abschließend wurden die erste und zweite Ableitung der Winkel pro Zeit, die physikalisch mit der Winkelgeschwindigkeit und der Winkelbeschleunigung korrespondieren, berechnet.

Für jeden der 30 Versuche wurden für das Einladen, die Fahrt und das Ausladen sowohl die Winkel – axiale Rotation, laterale Beugung und Flexion/Extension – als auch deren erste und zweite Ableitung betrachtet und für jeden Versuchsdurchlauf die Parameter mittlere Abweichung von der Nullposition, Größe des überstrichenen Winkelbereichs (Range) und Maximum berechnet. Mittels Mann-Whitney-U-Tests wurde überprüft, ob die Parameter im Vergleich mit und ohne die HWS-Orthese signifikante Unterschiede zeigen (zweiseitig, Signifikanzniveau 0,05). Außerdem wurde eine Power-Analyse durchgeführt (zweiseitig, Signifikanzniveau 0,05). Das 95 %-Konfidenzintervall der Differenz der Mittelwerte wurde über einen Fit mit einer Normalverteilung mit unterschiedlichen Varianzen geschätzt (SPSS Statistics, Version 29.0.1.0, IBM, Armonk, NY, USA).

## Ergebnisse

Ein kompletter Versuchsdurchlauf dauerte im Mittel (ohne die 30-sekündige Kalibrierung) 8,3 min (Standardabweichung ± 0,7, Minimum 7,4, Maximum 11,0), wobei auf den Vorgang des Einladens im Mittel 2,1 min (Standardabweichung ± 0,2, Minimum 1,8 und Maximum 2,7 min), auf die Fahrt 4,9 min (Standardabweichung ± 0,5, Minimum 4,2 und Maximum 6,8 min) und auf das Ausladen 1,4 min (Standardabweichung ± 0,2, Minimum 1,2 und Maximum 1,9 min) entfielen.

Wegen der Symmetrie des Kopfes wurden bei der axialen Rotation und der lateralen Beugung bei der Bestimmung von mittlerem Winkel und Maximum jeweils die Absolutwerte betrachtet, während bei der Bewegung in der Sagittalebene zwischen Flexion (nach vorn, positiver Winkel) und Extension (nach hinten, negativer Winkel) unterschieden wurde. Maximalwinkel, mittlere Winkel und Winkelbereiche für axiale Rotation, laterale Beugung und Flexion/Extension sind in den Tab. 2, 3 und 4 zusammengefasst.

**Tab. 2:** Axiale Rotation der Halswirbelsäule (HWS), mit ** gekennzeichnete Parameter beziehen sich auf die Absolutwerte; Einheit aller Werte ist Grad

		Mit HWS-Orthese		Ohne HWS-Orthese		Vergleich			
Zeitabschnitt	Parameter	Wert ± Standardabweichung	Minimum; Maximum	Wert ± Standardabweichung	Minimum; Maximum	*p*-Wert	Power	Differenz der Mittelwerte	95 %-Konfidenzintervall der Differenz der Mittelwerte
Einladen	Maximum**	0,68 ± 0,40	0,26; 1,75	0,84 ± 0,39	0,27; 1,66	0,187	0,187	−0,16	−0,45 bis 0,14
	Mittelwert**	0,44 ± 0,33	0,10; 1,32	0,52 ± 0,29	0,11; 1,04	0,285	0,106	−0,08	−0,32 bis 0,15
	Range	0,75 ± 0,38	0,36; 1,76	0,88 ± 0,36	0,44; 1,36	0,233	0,156	−0,13	−0,41 bis 0,15
Fahrt	Maximum**	0,90 ± 0,33	0,40; 1,58	1,26 ± 0,42	0,57; 1,94	0,100	0,723	−0,36	−0,64 bis −0,08
	Mittelwert**	0,46 ± 0,22	0,16; 0,92	0,70 ± 0,39	0,26; 1,50	0,074	0,517	−0,24	−0,48 bis 0,00
	Range	0,87 ± 0,22	0,40;1,23	1,07 ± 0,31	0,65; 1,78	0,148	0,492	−0,20	−0,40 bis 0,00
Ausladen	Maximum**	0,76 ± 0,35	0,37; 1,73	1,01 ± 0,44	0,43; 1,91	0,106	0,387	−0,25	−0,55 bis 0,46
	Mittelwert**	0,45 ± 0,24	0,22; 1,15	0,64 ± 0,33	0,20; 1,32	0,106	0,425	−0,20	−0,41 bis 0,23
	Range	0,71 ± 0,23	0,37; 1,18	0,82 ± 0,36	0,45; 1,74	0,512	0,167	−0,11	−3,41 bis 0,11

**Tab. 3:** Laterale Beugung der Halswirbelsäule (HWS), mit ** gekennzeichnete Parameter beziehen sich auf die Absolutwerte; Einheit aller Werte ist Grad

		Mit HWS-Orthese		Ohne HWS-Orthese		Vergleich			
Zeitabschnitt	Parameter	Wert ± Standardabweichung	Minimum; Maximum	Wert ± Standardabweichung	Minimum; Maximum	*p*-Wert	Power	Differenz der Mittelwerte	95 %-Konfidenzintervall der Differenz der Mittelwerte
Einladen	Maximum**	2,11 ± 1,36	0,65; 4,62	1,41 ± 0,71	0,32; 2,47	0,325	0,386	0,69	−0,13 bis 1,52
	Mittelwert**	1,15 ± 0,98	0,30; 3,05	0,79 ± 0,53	0,06; 1,65	0,539	0,225	0,36	−0,24 bis 0,96
	Range	2,13 ± 1,31	0,82; 4,55	1,44 ± 0,55	0,47; 2,50	0,325	0,435	0,69	−0,74 bis 1,46
Fahrt	Maximum**	3,32 ± 2,19	0,81; 7,99	2,41 ± 1,13	0,79; 4,17	0,367	0,193	0,91	−0,41 bis 2,23
	Mittelwert**	2,42 ± 2,24	0,40; 7,01	1,68 ± 1,17	0,26; 3,54	0,653	0,192	0,74	−0,61 bis 2,10
	Range	2,55 ± 1,20	1,18; 5,02	2,13 ± 0,82	1,02; 3,38	0,367	0,186	0,41	−0,36 bis 1,19
Ausladen	Maximum**	3,08 ± 1,84	0,89; 6,67	2,12 ± 0,79	0,86; 3,09	0,285	0,417	0,96	−0,13 bis 2,04
	Mittelwert**	2,26 ± 1,69	0,36; 5,85	1,43 ± 0,75	0,46; 2,38	0,325	0,382	0,83	−0,16 bis 1,83
	Range	1,56 ± 0,66	0,61; 2,83	1,58 ± 0,64	0,82; 2,82	0,967	0,050	−0,01	−0,49 bis 0,48

**Tab. 4:** Flexion/Extension der Halswirbelsäule (HWS) in der Sagittalebene, mit * gekennzeichnete *p*-Werte weisen auf einen signifikanten Unterschied der zugehörigen Parameter zwischen den Experimenten mit und ohne HWS-Orthese hin; Einheit aller Werte ist Grad

		Mit HWS-Orthese		Ohne HWS-Orthese		Vergleich			
Zeitabschnitt	Parameter	Wert ± Standardabweichung	Minimum; Maximum	Wert ± Standardabweichung	Minimum; Maximum	*p*-Wert	Power	Differenz der Mittelwerte	95 %-Konfidenzintervall der Differenz der Mittelwerte
Einladen	Maximum Extension	−0,47 ± 0,34	−1,04; 0,20	−0,41 ± 0,21	−0,91; −0,18	0,595	0,910	−0,06	−0,28 bis 0,15
	Maximum Flexion	0,19 ± 0,22	−0,09;0,71	0,41 ± 0,38	0,38; −0,07	0,137	0,428	−0,21	−0,45 bis 0,02
	Mittelwert	−014 ± 0,34	−0,72; 0,52	0,01 ± 0,32	−0,62; 0,54	0,217	0,192	−0,14	−0,39 bis 0,10
	Range	0,66 ± 0,19	0,30; 1,01	0,81 ± 0,33	0,40; 1,50	0,412	0,308	−015	−0,35 bis 0,05
Fahrt	Maximum Extension	−1,09 ± 0,83	−2,53; −0,08	−0,40 ± 0,58	−1,59; 0,29	0,160	0,717	−0,69	−1,23 bis −0,15
	Maximum Flexion	0,00 ± 0,53	−0,95; 0,98	0,64 ± 0,39	−0,20; 1,10	0,001*	0,953	−0,64	−0,99 bis −0,29
	Mittelwert	−0,57 ± 0,73	−1,81; 0,58	0,11 ± 0,43	−0,73; 0,67	0,007*	0,842	−0,68	−1,13 bis −0,22
	Range	1,09 ± 0,50	0,36; 2,10	1,04 ± 0,40	0,64; 2,01	0,683	0,060	0,05	−0,29 bis 0,39
Ausladen	Maximum Extension	−1,06 ± 0,89	−2,55; 0,38	−0,48 ± 0,61	−1,64; 0,40	0,061	0,516	−0,58	−1,15 bis −0,01
	Maximum Flexion	−0,32 ± 0,85	−1,67; 1,28	0,37 ± 0,61	−0,44; 1,62	0,041*	0,693	−0,69	−1,24 bis −0,14
	Mittelwert	−0,68 ± 0,86	−2,31; 0,80	−0,06 ± 0,47	−0,95; 0,55	0,050*	0,645	−0,62	−1,15 bis −0,09
	Range	0,74 ± 0,40	0,23; 1,41	0,85 ± 0,44	0,26; 1,77	0,683	0,106	−0,11	−0,42 bis 0,20

**Abb. 3:**
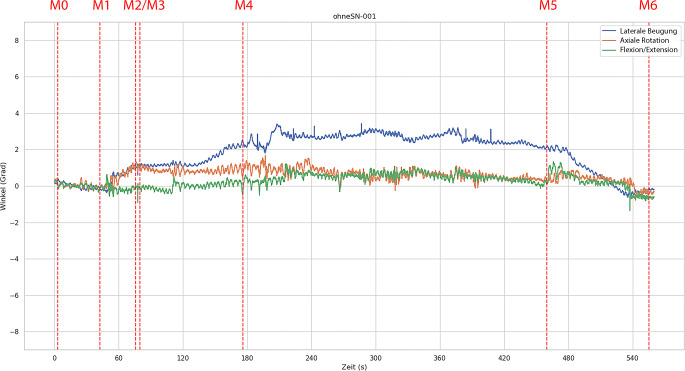
Exemplarischer Plot der gemessenen Winkelauslenkungen bei einem Versuchsablauf ohne starre Halswirbelsäulen-Orthese

**Abb. 4:**
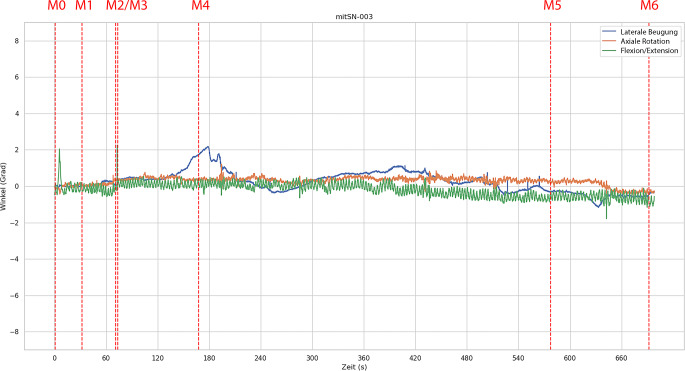
Exemplarischer Plot der gemessenen Winkelauslenkungen bei einem Versuchsablauf mit starrer Halswirbelsäulen-Orthese

Abb. 3 zeigt beispielhaft die Winkel in allen 3 Dimensionen über die Zeit für einen der 15 Versuchsdurchläufe ohne starre HWS-Orthese, Abb. 4 korrespondierend eine exemplarische Messung mit starrer HWS-Orthese.

## Diskussion

Die Immobilisation der HWS mittels starrer Orthese sollte aufgrund potenzieller, durch diese Maßnahme bedingter Probleme wie Ansteigen des intrakraniellen Drucks oder Beeinträchtigung der oberen Atemwege und erschwerte Bedingungen bei der Atemwegssicherung [3, 5–8, 11] nicht grundsätzlich und nicht unkritisch erfolgen.

Die aktuell gültige deutsche S3-Leitlinie „Polytrauma/Schwerverletzten-Behandlung“ empfiehlt noch die Stabilisierung der HWS vor der eigentlichen technischen Rettung, außer in Fällen, in denen eine sofortige Rettung des Patienten unabdingbar ist, wie bei Bränden oder Explosionsgefahr [2]. Es wird jedoch betont, dass es keinen wirklichen Beweis für einen positiven Effekt der Stabilisierung gibt. Aus grundsätzlichen Überlegungen sollte es vorteilhaft sein, die Zeitdauer, während derer eine starre HWS-Orthese angelegt ist, in der prähospitalen Phase so kurz wie möglich zu halten. In einer ersten Versuchsreihe unter Verwendung der gleichen Messtechnik konnten wir bei der simulierten Umlagerung eines am Boden liegenden Traumapatienten auf eine Trage unter Verwendung gängiger Hilfsmittel feststellen, dass eine starre HWS-Orthese Bewegungen im Bereich der HWS im Vergleich zur analogen Versuchsdurchführung ohne HWS-Orthese deutlich reduzierte [9]. In der vorliegenden Arbeit sollte nun die darauffolgende Phase der prähospitalen Versorgung untersucht und hinterfragt werden, ob die Beibehaltung der Immobilisation der HWS mittels starrer Orthese noch einen zusätzlichen Vorteil im Hinblick auf die Einschränkung der Beweglichkeit der HWS bringt, sobald der Patient unter Verwendung einer Vakuummatratze mit der Möglichkeit zur seitlichen Fixierung des Kopfes auf der Trage immobilisiert gelagert ist.

Bei der Betrachtung des zeitlichen Verlaufs der gemessenen Winkel und deren erster und zweiter Ableitung (nicht abgebildet) sieht man, dass sowohl das Antippen des Stirnsensors vor dem Einladen der Trage in den RTW, der Vorgang des Ein- und Ausladens der Trage und auch Besonderheiten der Strecke während der Fahrt (insbesondere die erste Kurve beim Einbiegen von der Zufahrt der Garage zur Straße) jeweils ihr Korrelat in größeren gemessenen Winkeln zu diesen Zeitpunkten finden und die Plausibilität der durchgeführten Messungen belegen.

Statistisch signifikante Unterschiede zwischen den betrachteten Parametern zur Abbildung der Bewegung der HWS ergaben sich allerdings nur bei einigen Parametern in der sagittalen Ebene, also bei Flexion und Extension. Für die anderen Bewegungsrichtungen (axiale Rotation, laterale Beugung) wurden keine signifikanten Unterschiede für die gemessenen Parameter ermittelt. Grundsätzlich liegen alle Mittelwerte der betrachteten Parameter bei axialer Rotation und Flexion/Extension in Bereichen von einem bis 2 Grad, bei der lateralen Bewegung bei bis zu 3 Grad, also einem Umfang, der eine klinische Relevanz fraglich erscheinen lässt. Bisher wurden allerdings keine Daten dazu publiziert, welches Ausmaß an Bewegung der HWS eine sekundäre Schädigung zervikalen Rückenmarks bei Vorliegen einer Verletzung der HWS verursachen kann.

Unter Verwendung des gleichen Messsystems wurden von Nolte et al. bereits Versuche während des Transports eines Probanden in einem RTW entlang einer vordefinierten Strecke durchgeführt [10]. Die Messungen erfolgten hierbei vergleichend unter Verwendung eines Spineboards oder einer Vakuummatratze mit und ohne starre HWS-Orthese. Zur Bewertung der gemessenen Bewegungen wurde von den Autoren unter der Annahme, dass eine kleinere, langsam ablaufende Winkelbewegung weniger wahrscheinlich zu einer sekundären Schädigung des zervikalen Rückenmarks führt als eine große, schnell ablaufende, ein Motionscore unter Berücksichtigung der Parameter Zeit und Winkelbewegung definiert und berechnet. Betrachtet man hier die Messungen, die auf einer Vakuummatratze mit seitlicher Stabilisierung des Kopfes mit und ohne starre HWS-Orthese durchgeführt wurden, ergaben sich in Übereinstimmung zu unseren Messungen keine relevanten Unterschiede in allen betrachteten Bewegungsrichtungen im Hinblick auf den ermittelten Motionscore.

Eine gewichtige Limitation unserer Versuche ist der Tatsache geschuldet, dass die Probandin wach war und möglicherweise durch ein unbewusstes Anspannen der Halsmuskulatur Bewegungen, die bei einem bewusstlosen oder analgosedierten und ggf. auch muskelrelaxierten Patienten ansonsten aufgetreten wären, entgegenwirkte. Als weitere Limitation muss erwähnt werden, dass alle Versuche mit nur einer Probandin und von immer demselben Team durchgeführt wurden.

## Fazit für die Praxis

Bei einer korrekt durchgeführten Immobilisation mittels einer Vakuummatratze mit der Möglichkeit zur seitlichen Stabilisierung des Kopfes sowie Kinn- und Stirngurt auf einer elektrohydraulischen Fahrtrage mit Beladesystem und luftgefederter Tragenlagerung ergeben sich für den Ein- und Ausladeprozess sowie während der Fahrt in einem modernen RTW mit Luftfederung der Hinterachse keine relevanten Vorteile bezüglich der Einschränkung der Bewegung der HWS durch die zusätzliche Verwendung einer starren HWS-Orthese. Es wäre daher denkbar, die zunächst für die Rettung des Patienten an der Einsatzstelle angelegte starre HWS-Orthese nach der Lagerung des Patienten auf der Vakuummatratze und Trage für den Zeitraum des Transports ins Krankenhaus wieder abzunehmen, um so potenzielle negative Effekte durch die starre HWS-Orthese zu vermeiden.

## Data Availability

Die den Berechnungen zugrunde liegenden Daten können bei den Autoren angefordert und eingesehen werden.
